# Supplementary material for: Bioinformatic Analysis of Structure and Function of LIM Domains of Human Zyxin Family Proteins
Source: Int J Mol Sci. 2021 Mar 5;22(5):2647. doi: 10.3390/ijms22052647 (PMC7961639; doi:10.3390/ijms22052647)
Supplement: Supplementary file 1 [file ijms-22-02647-s001.pdf]

Supplementary Materials:

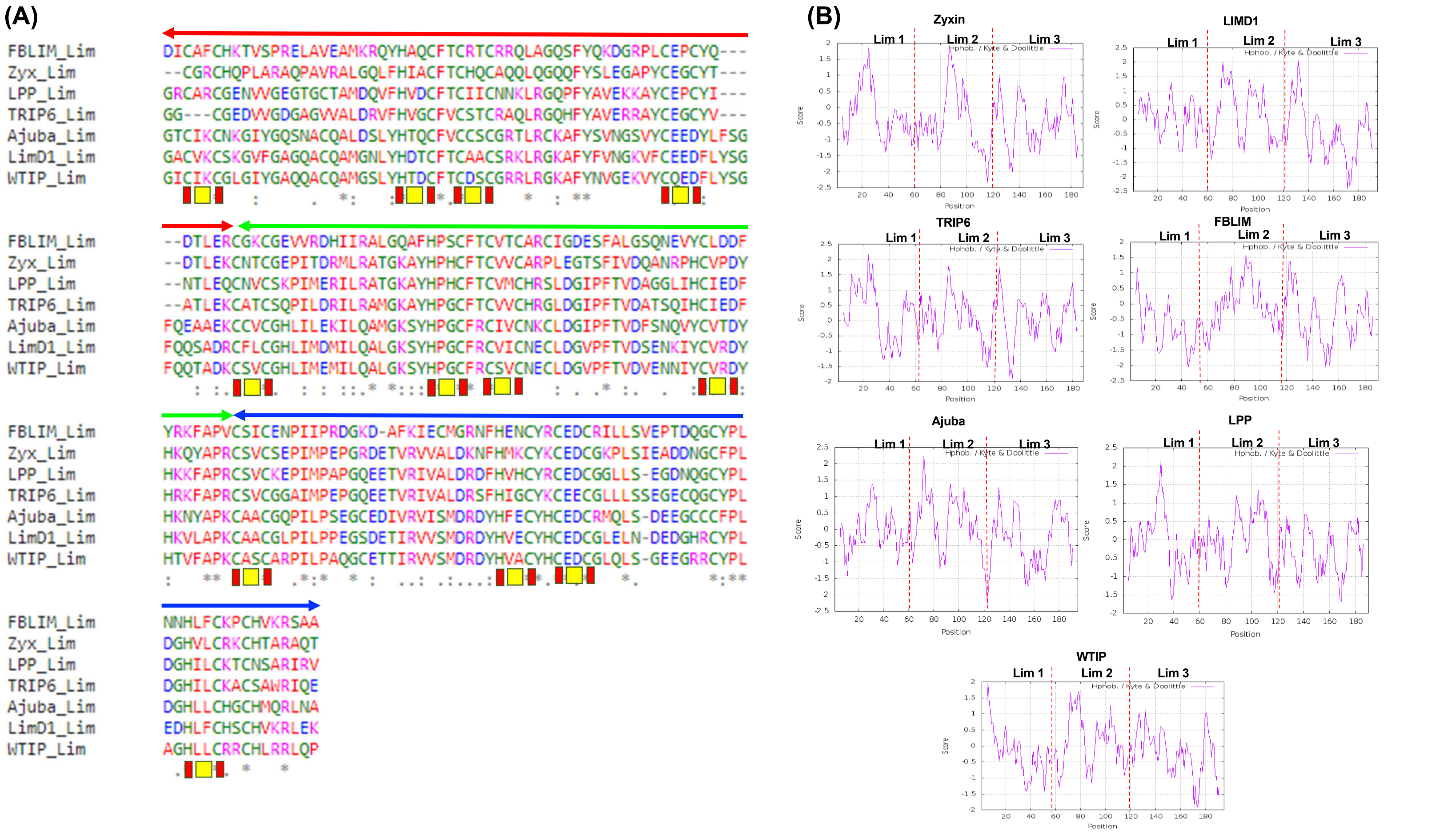

Supplementary Figure 1. (A) MUSCLE sequence alignment of the Zyxin family Lim domain shows the presence of conserved cysteine/Histidine residues in Lim1 (red arrow)/Lim2 (green arrow)/Lim3(blue arrow) regions (red boxes are conserved cysteine/histidine/aspartate, Yellow boxes are variable hydrophobic residues) (B) Hydrophobicity plots for Lim domains of Zyxin family proteins. The hydrophobicity analysis was performed using the ProtScale server and plotted against the amino acid position. The scoring from -2.0 to 2.5 on the y-axis reflects hydrophobicity (higher positive (+ve) score depicts more hydrophobicity). This analysis indicates that each Lim domain and Lim1/Lim2/Lim3 region has a distinct hydrophobicity score.

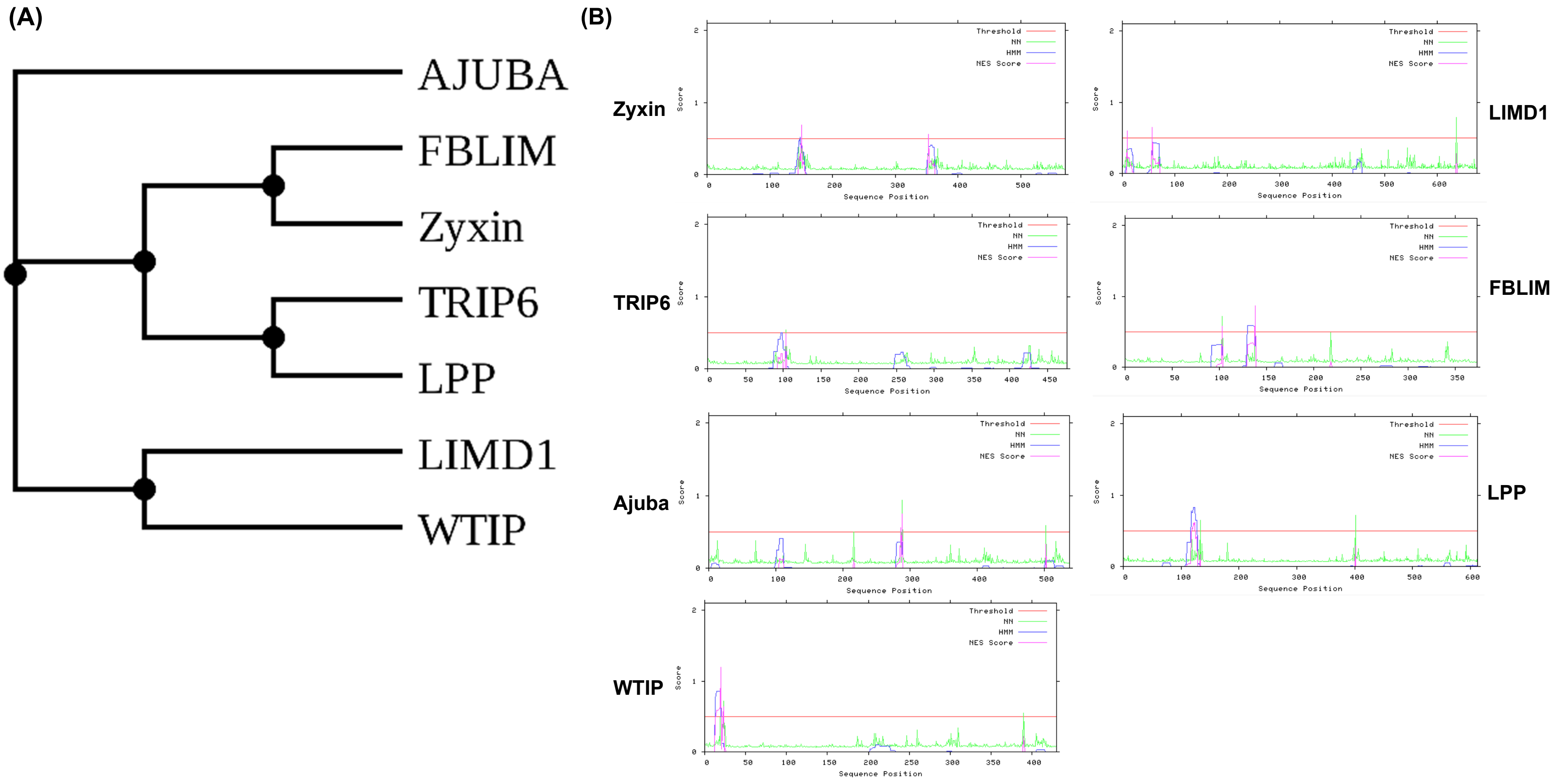

Supplementary Figure 2. Phylogenetic and NetNES analysis of the Zyxin family proteins. (A) phylogenetic tree created by Maxximum Likelihoos Method on PhyML 3.0 server (B) NetNES analysis was done to identify the Leucine-rich motif nuclear export sequence (NES) responsible for nuclear transport of protein. The NES was identified through combined analysis implemented by Neural Network (NN), Hidden Markov Model (HMM) and NES scoring algorithm. Only the sequences giving a peak higher than threshold using all three algorithms was identified as NES.

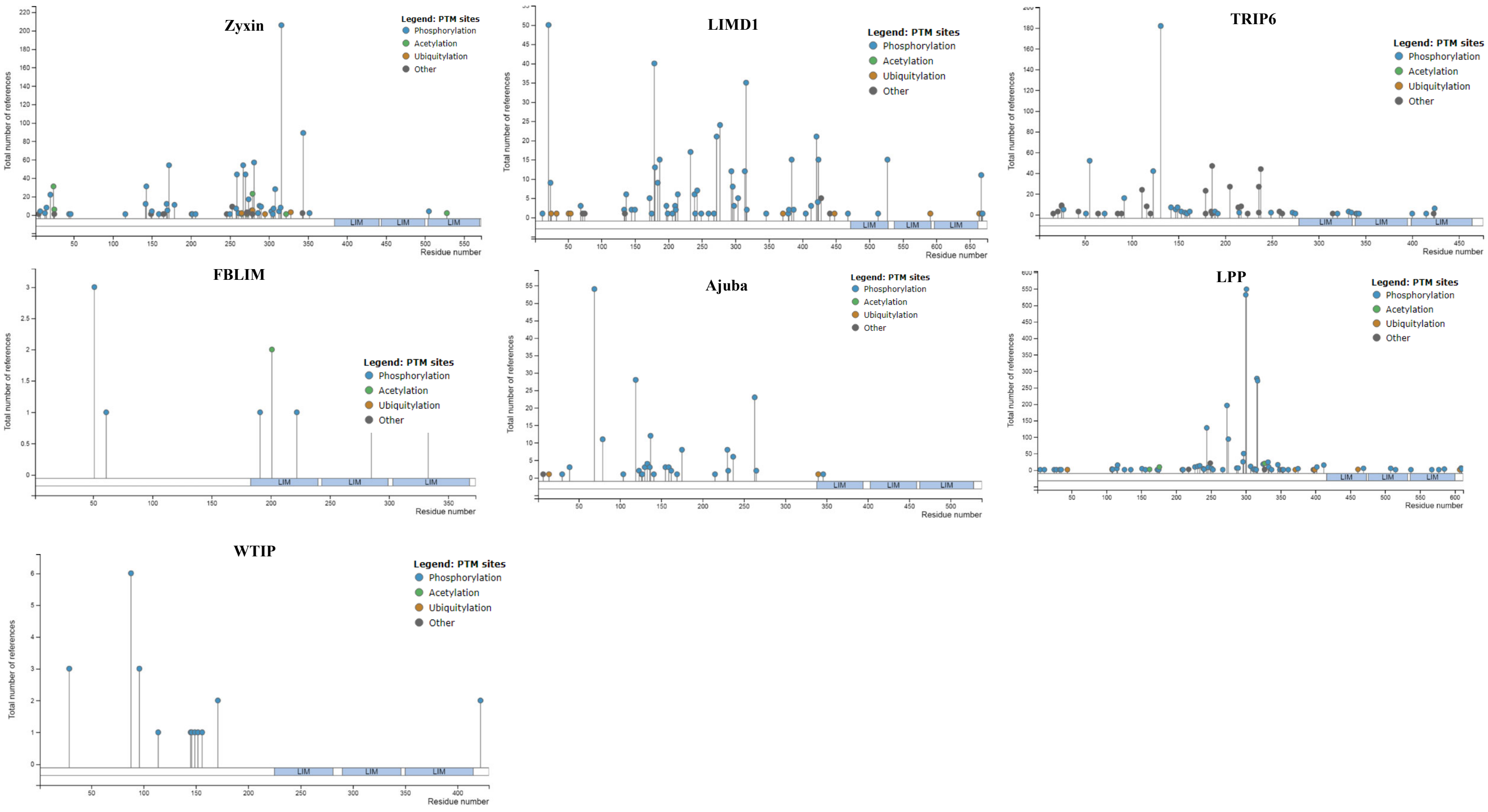

Supplementary Figure 3. Identification of post-translation modification (PTM) sites of Zyxin family proteins. All proteins exhibited sites for PTM. However, PTMs varied amongst all the member proteins, with the highest PTM observed for LPP, and the lowest for FBLIM1.

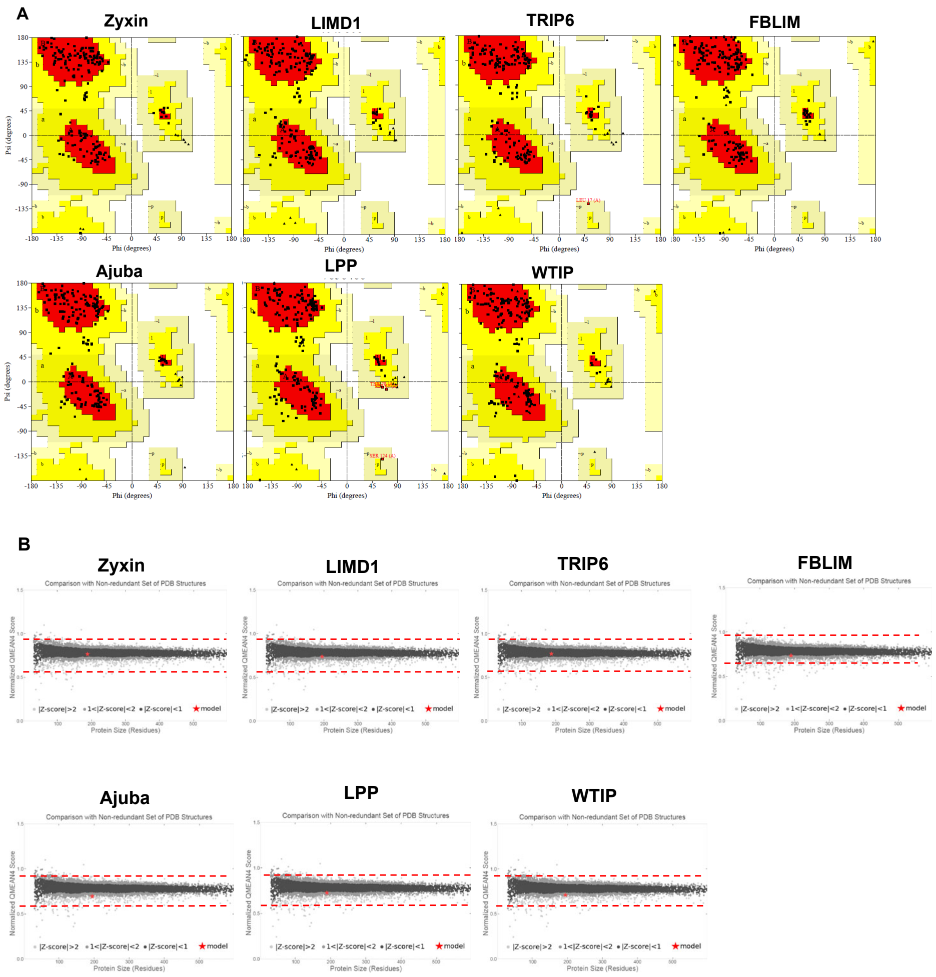

Supplementary Figure 4. (A) Validation of homology models by Ramachandran plot. Models generated for members of Zyxin family were validated through Ramachandran plots. All models had acceptable Ramachandran values. (B) Absolute quality estimation of all the models by QMEAN, reflecting the native character and quality, red star indicating the query model position while comparing with all available models in PDB.

```
# Job: Lim_Zyx
# Query: s001A
# No:  Chain   Z    rmsd lali nres  %id PDB  Description
   1:  1rut-X  9.7  4.7  141   157   26   MOLECULE: FUSION PROTEIN OF LMO4 PROTEIN AND LIM DOMAIN-BIN
   2:  2dfy-X  9.5  4.6  140   158   26   MOLECULE: FUSION PROTEIN OF LIM DOMAIN TRANSCRIPTION FACTOR
   3:  2dfy-C  9.4  4.6  140   158   26   MOLECULE: FUSION PROTEIN OF LIM DOMAIN TRANSCRIPTION FACTOR
   4:  2rgt-B  9.4  7.1  135   154   28   MOLECULE: FUSION OF LIM/HOMEOBOX PROTEIN LHX3, LINKER, INSU
   5:  6cme-A  9.4 13.2   98   154   24   MOLECULE: LIM/HOMEOBOX PROTEIN LHX4,INSULIN GENE ENHANCER P
   6:  4jcj-B  9.4  4.1   86   148   23   MOLECULE: INSULIN GENE ENHANCER PROTEIN ISL-1,LIM DOMAIN-BI
   7:  3mmk-A  9.3 12.9  118   151   30   MOLECULE: FUSION OF LIM/HOMEOBOX PROTEIN LHX4, LINKER, INSU
   8:  4jcj-C  9.2  4.4   86   150   23   MOLECULE: INSULIN GENE ENHANCER PROTEIN ISL-1,LIM DOMAIN-BI
   9:  4jcj-A  9.2  3.7   81   144   25   MOLECULE: INSULIN GENE ENHANCER PROTEIN ISL-1,LIM DOMAIN-BI
  10:  3mmk-B  9.2 13.2  121   154   28   MOLECULE: FUSION OF LIM/HOMEOBOX PROTEIN LHX4, LINKER, INSU
  11:  2rgt-A  8.5  4.9  118   148   30   MOLECULE: FUSION OF LIM/HOMEOBOX PROTEIN LHX3, LINKER, INSU
  12:  1b8t-A  7.5  2.0   65   192   26   MOLECULE: PROTEIN (CRP1);
  13:  2miu-A  7.3  4.6   75    98   24   MOLECULE: FOUR AND A HALF LIM DOMAINS PROTEIN 2;
  14:  2xjz-E  7.1  6.1  115   127   25   MOLECULE: RHOMBOTIN-2;
  15:  2xjz-D  7.0  5.9  115   129   25   MOLECULE: RHOMBOTIN-2;
  16:  2xjy-A  7.0  1.9   59   131   27   MOLECULE: RHOMBOTIN-2;
  17:  2dlo-A  6.8  2.5   64    81   59   MOLECULE: THYROID RECEPTOR-INTERACTING PROTEIN 6;
  18:  2xqn-T  6.8  2.3   60   125   28   MOLECULE: ACTIN-LIKE PROTEIN 7A;
  19:  2ypa-C  6.8  1.8   56   126   27   MOLECULE: T-CELL ACUTE LYMPHOCYTIC LEUKEMIA PROTEIN 1;
  20:  2lzu-A  6.8  2.4   64    72   22   MOLECULE: LIM DOMAIN-CONTAINING PROTEIN 2;
  21:  2xjz-B  6.8  5.2  113   122   25   MOLECULE: RHOMBOTIN-2;
  22:  2iyb-H  6.7  2.8   58    63   21   MOLECULE: PROTEIN ENABLED HOMOLOG;
  23:  2iyb-G  6.7  2.9   59    64   20   MOLECULE: PROTEIN ENABLED HOMOLOG;
  24:  2iyb-F  6.7  3.0   58    63   21   MOLECULE: PROTEIN ENABLED HOMOLOG;
  25:  1x61-A  6.6  3.5   63    72   25   MOLECULE: THYROID RECEPTOR INTERACTING PROTEIN 6;
  26:  4kfz-A  6.6  8.3  109   146   25   MOLECULE: LMO-2;
  27:  2xjz-A  6.6  5.9  111   125   24   MOLECULE: RHOMBOTIN-2;
  28:  2mbv-A  6.5  3.8   72    96   26   MOLECULE: FUSION PROTEIN OF LIM DOMAIN TRANSCRIPTION FACTOR
  29:  2iyb-E  6.5  3.1   59    64   20   MOLECULE: PROTEIN ENABLED HOMOLOG;
  30:  2xjz-C  6.5  3.1   60   112   17   MOLECULE: RHOMBOTIN-2;
  31:  6cme-B  6.4 13.2  122   154   28   MOLECULE: LIM/HOMEOBOX PROTEIN LHX4,INSULIN GENE ENHANCER P
  32:  2dar-A  6.3  2.2   62    90   26   MOLECULE: PDZ AND LIM DOMAIN PROTEIN 5;
  33:  2dj7-A  6.3  7.9   72    80   28   MOLECULE: ACTIN-BINDING LIM PROTEIN 3;
```

Supplementary Figure 5. Structural alignment analysis of the Zyxin Lim domain by DALI server, indicates the similarity with transcription regulators.

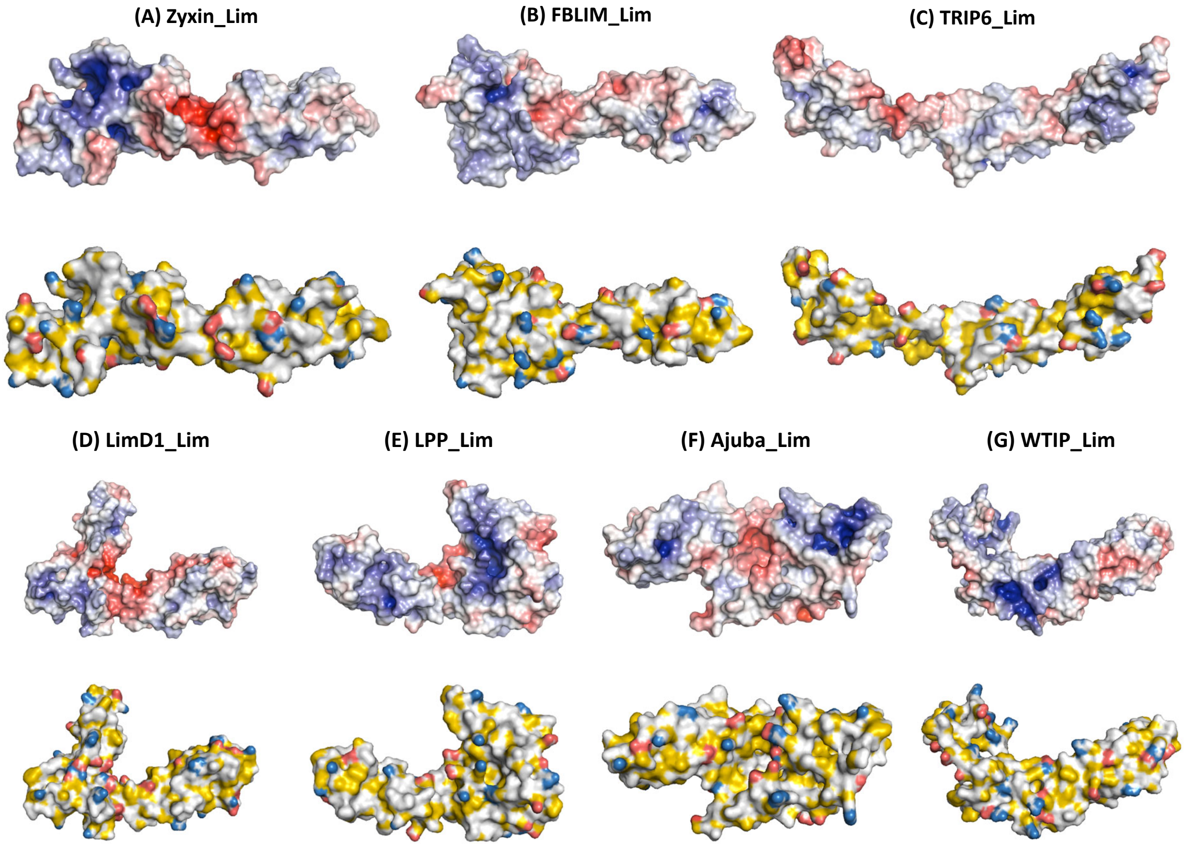

Supplementary Figure 6. (A) Adaptive Poisson-Boltzmann Solver (APBS) electrostatics and Yellow-Red-Blue (YRB) showing surface potential and hydrophobicity calculations, respectively; of Zyxin family Lim domains.

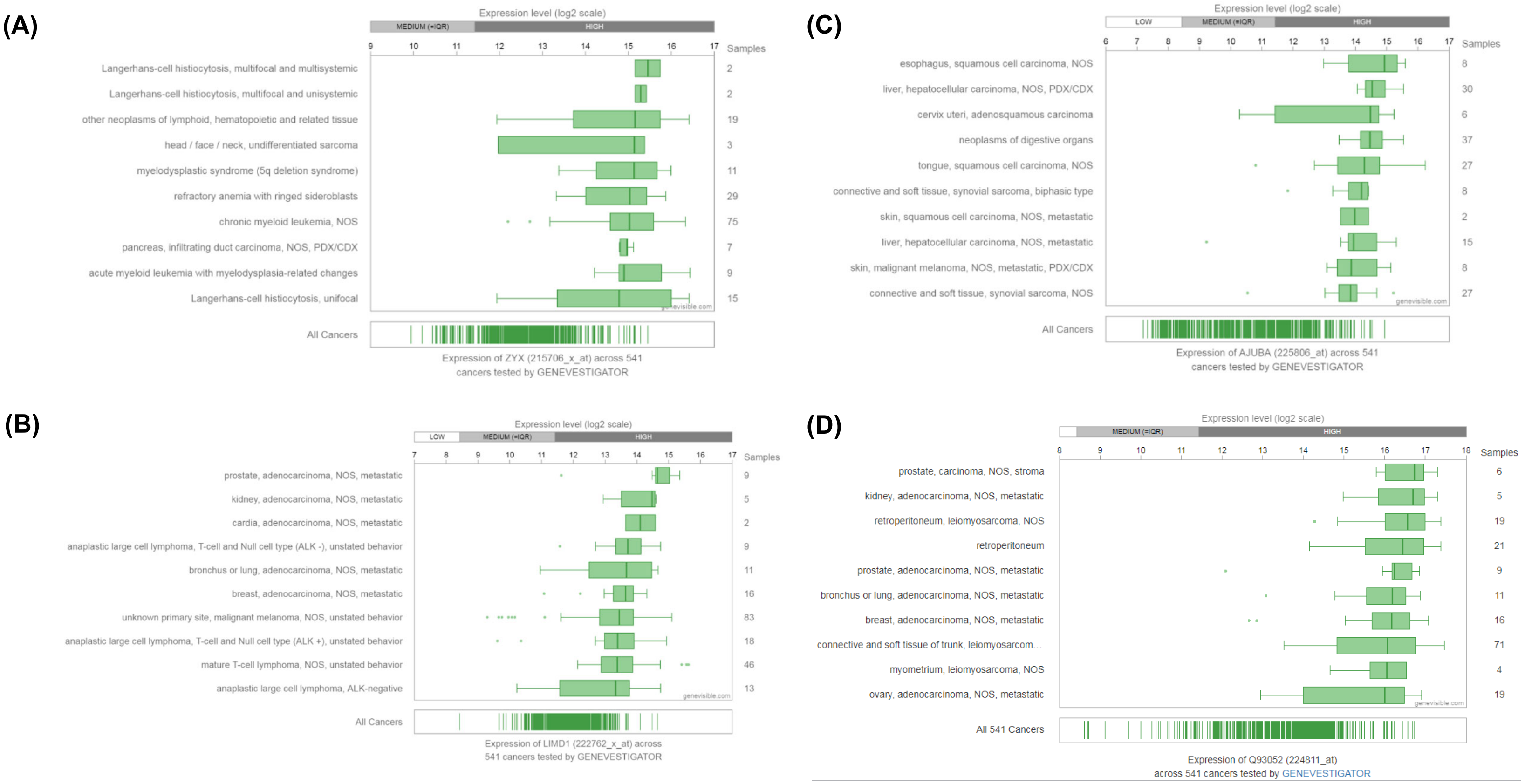

Supplementary Figure 7. The protein expression profiles of the Zyxin family proteins in different cancers (retrieved from Genevestigator) are presented as bar graphs. (A) Zyxin; (B) LIMD1; (C) Ajuba and (D) LPP. Evidently, the expression of Zyxin family proteins was high in almost all types of cancers.

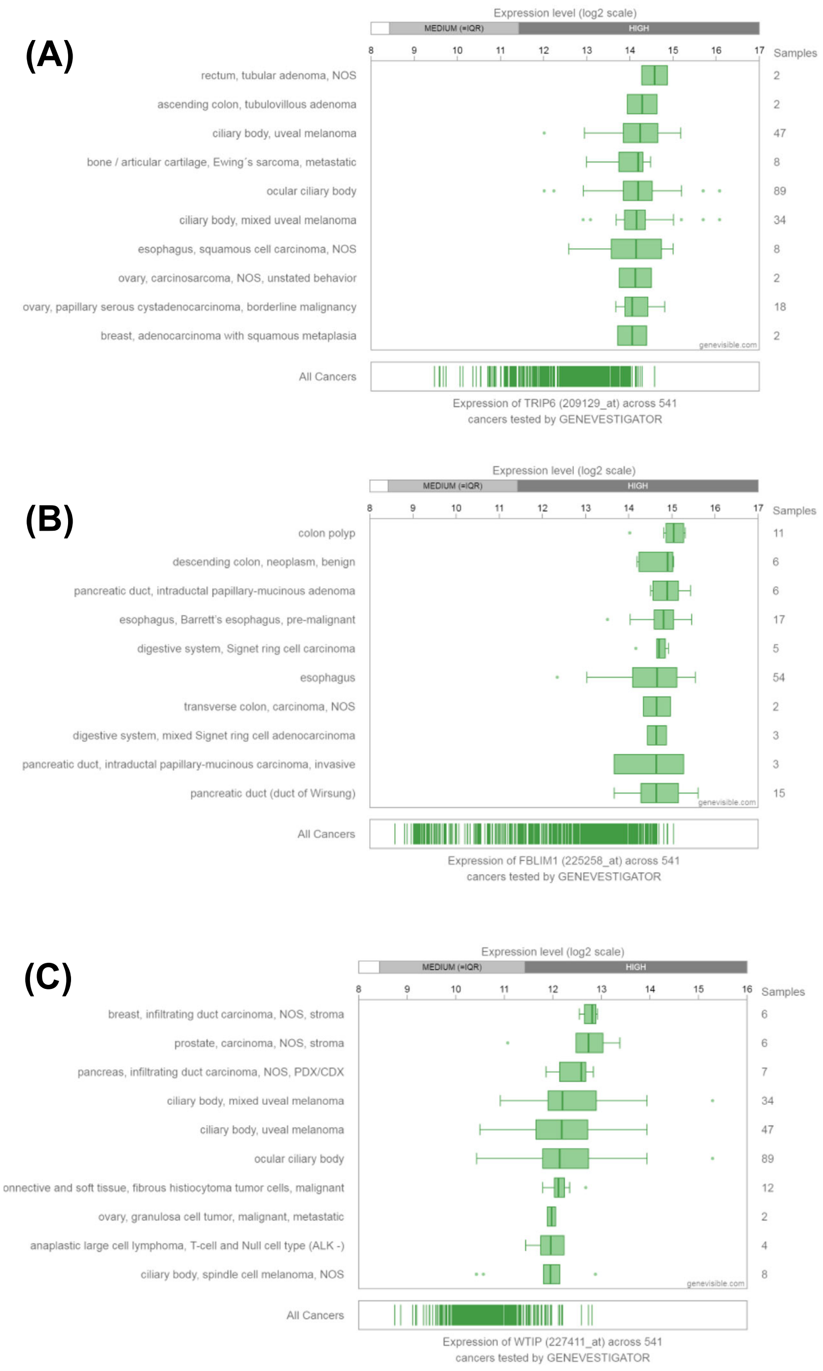

Supplementary Figure 8. The protein expression profiles of the Zyxin family proteins in different cancers (retrieved from Genevestigator) are presented as bar graphs. (A) TRIP6; (B) FBLIM; (C) WTIP. Evidently, the expression of Zyxin family proteins was high in almost all types of cancers.

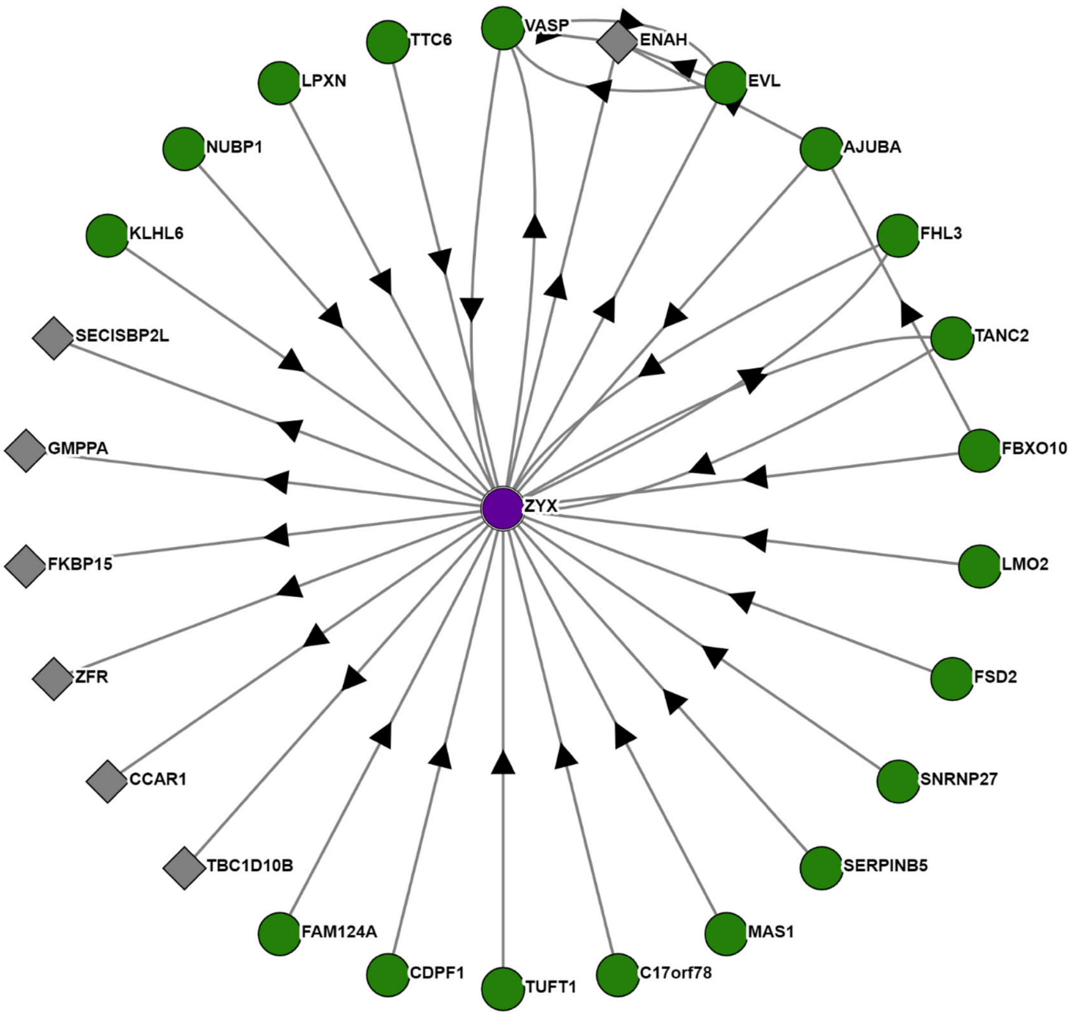

Supplementary Figure 9. Protein-protein interaction (PPI) analysis of Zyxin. The arrow indicates the bait and prey relationship of each protein. The bait protein is shown as a green circle whereas the prey protein is shown as grey squares.

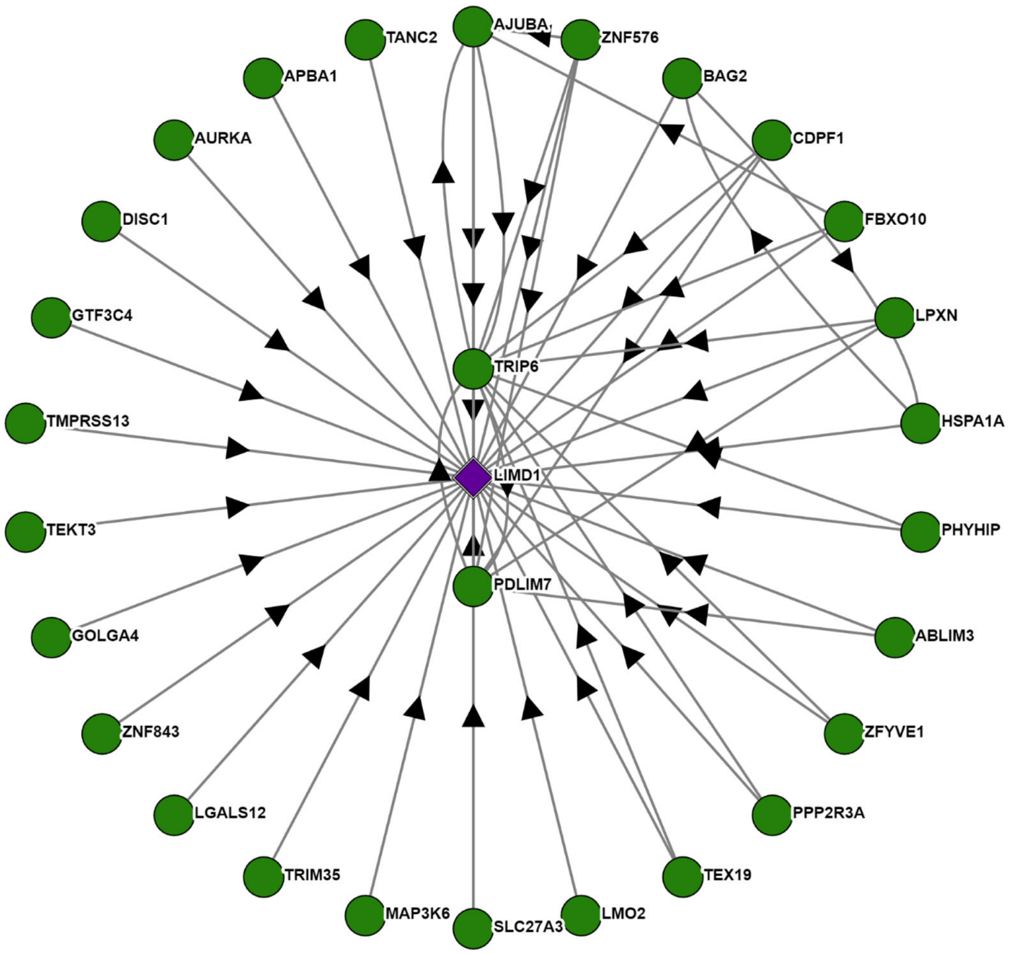

Supplementary Figure 10. Protein-protein interaction (PPI) analysis of LimD1. The arrow indicates the bait and prey relationship of each protein. The bait protein is shown as a green circle whereas the prey protein is shown as grey squares.

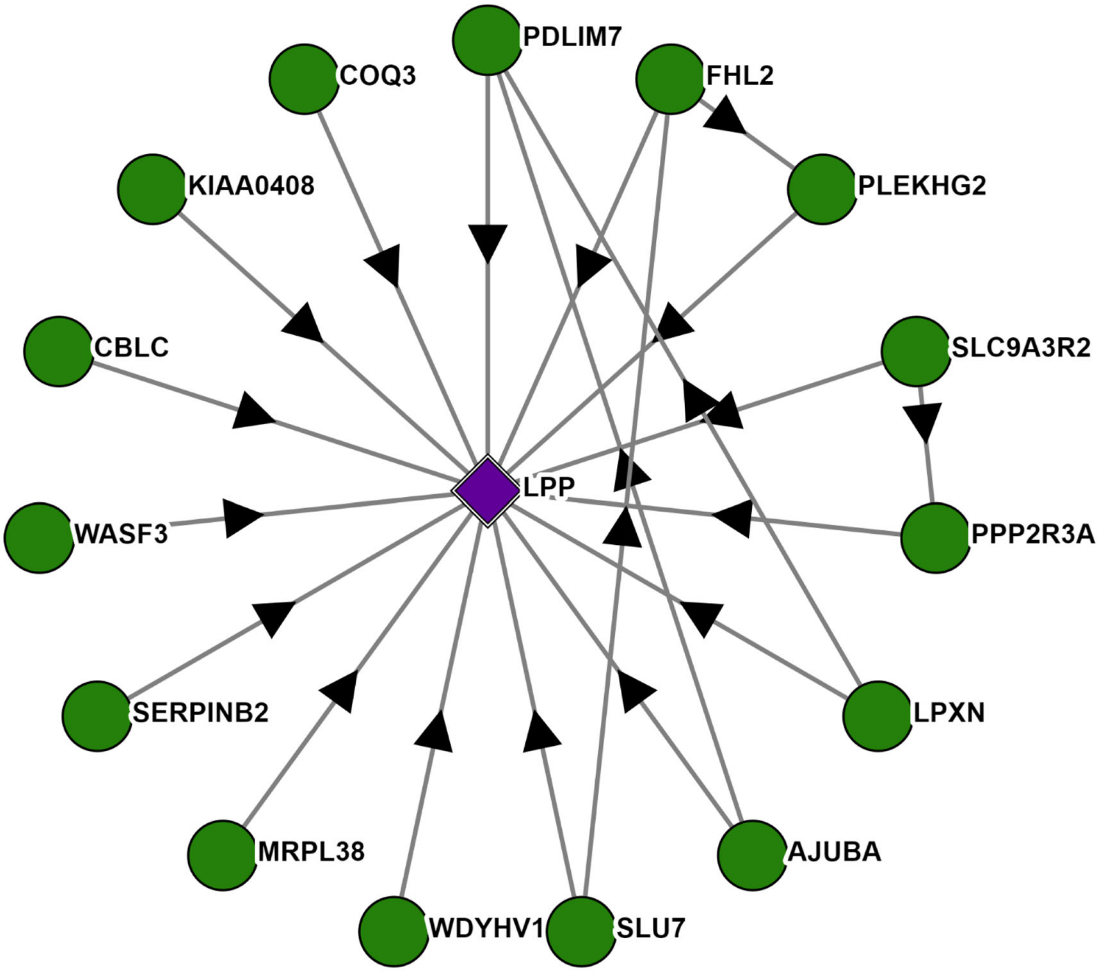

Supplementary Figure 11. Protein-protein interaction (PPI) analysis of LPP. The arrow indicates the bait and prey relationship of each protein. The bait protein is shown as a green circle whereas the prey protein is shown as grey squares.

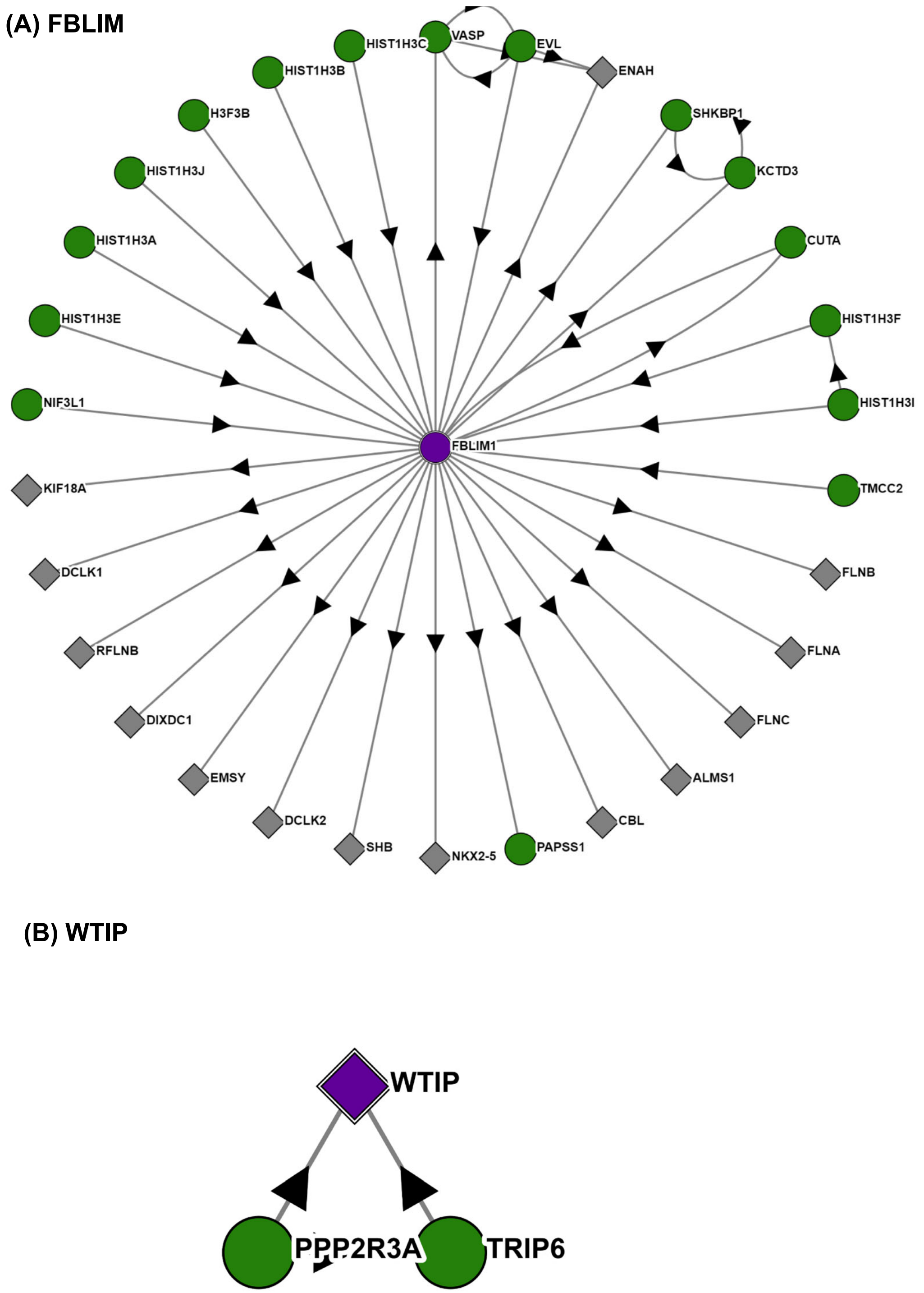

Supplementary Figure 12. Protein-protein interaction (PPI) analysis of (A) FBLIM and (B) WTIP. The arrow indicates the bait and prey relationship of each protein. The bait protein is shown as a green circle whereas the prey protein is shown as grey squares. The PPI analysis suggests WTIP has the least interacting partners.

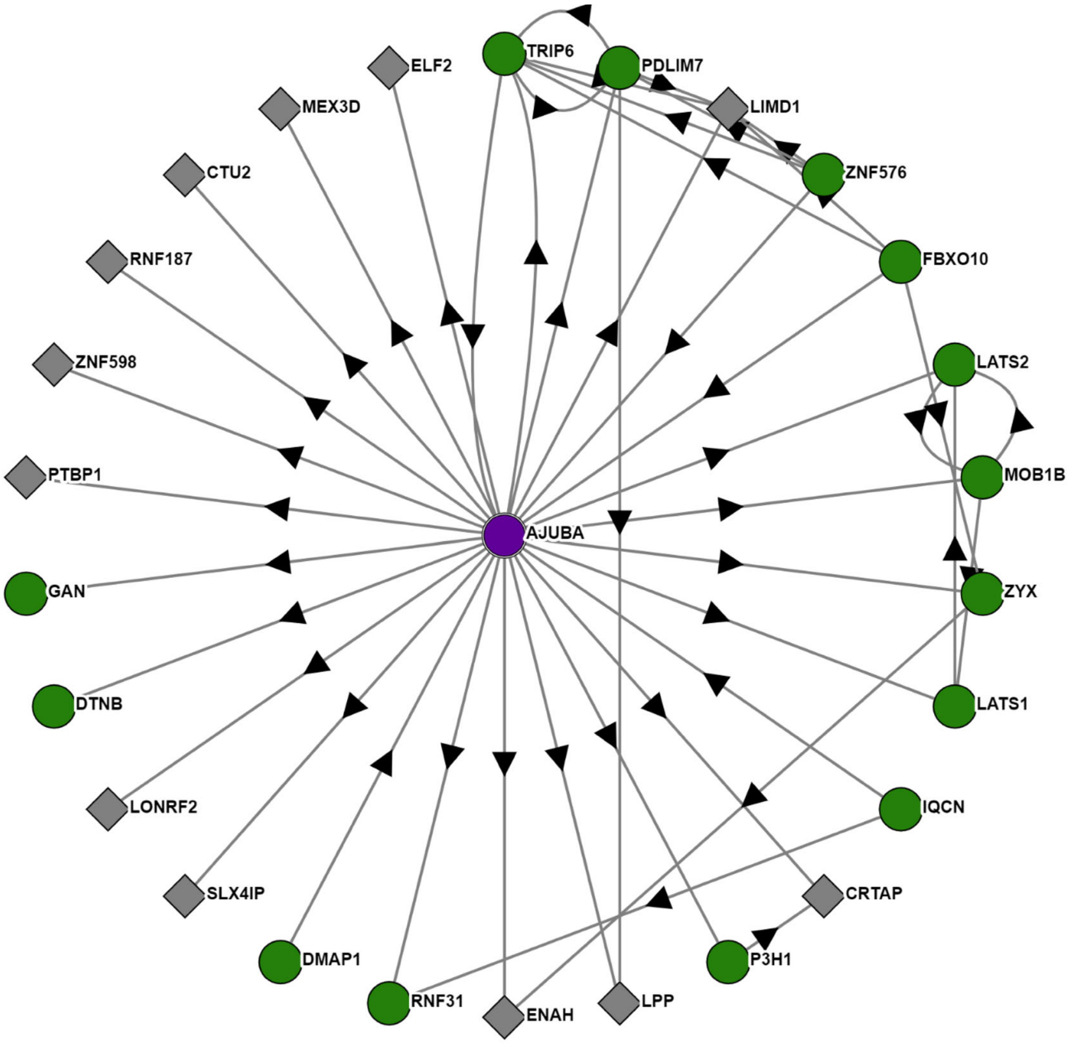

Supplementary Figure 13. Protein-protein interaction (PPI) analysis of Ajuba. The arrow indicates the bait and prey relationship of each protein. The bait protein is shown as a green circle whereas the prey protein is shown as grey squares.

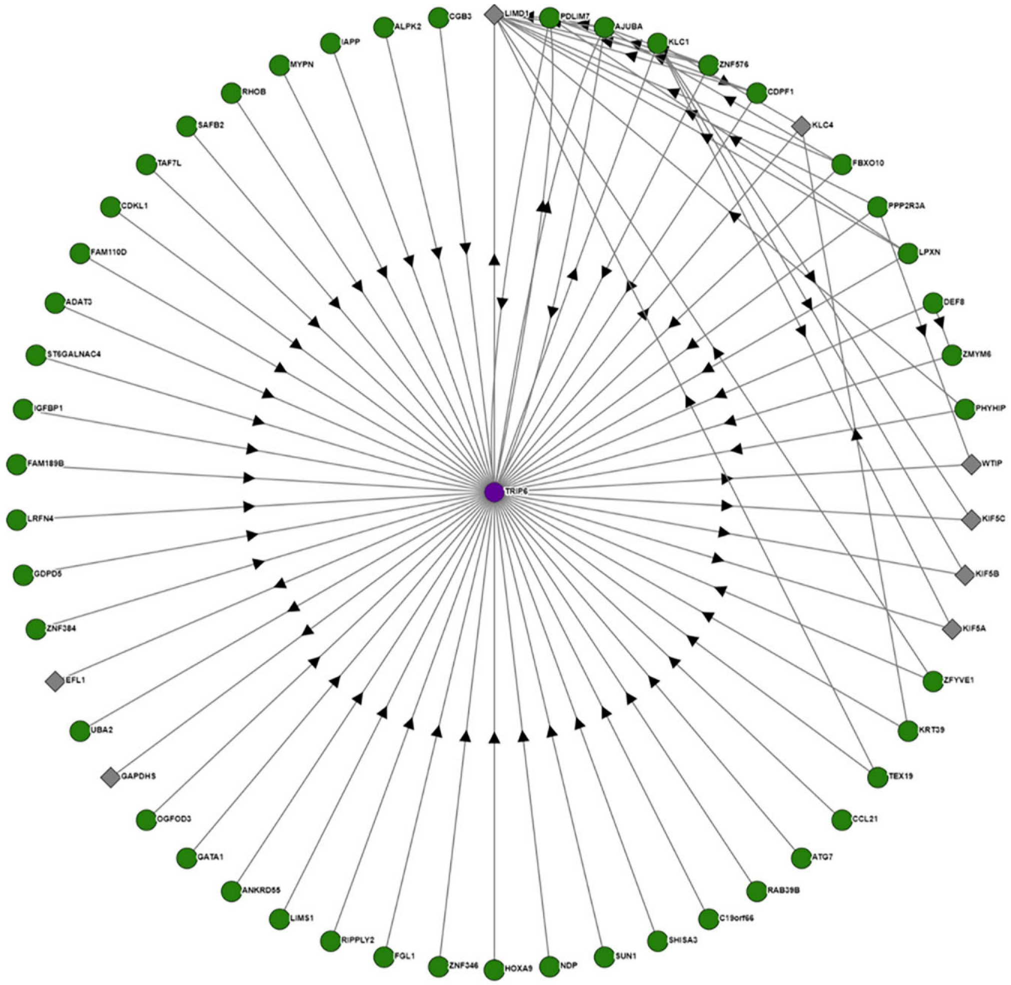

Supplementary Figure 14. Protein-protein interaction (PPI) analysis of TRIP6. The arrow indicates the bait and prey relationship of each protein. The bait protein is shown as a green circle whereas the prey protein is shown as grey squares. The PPI analysis suggests that TRIP6 has the maximum number of interacting partners.

(A) OmniPath Analysis

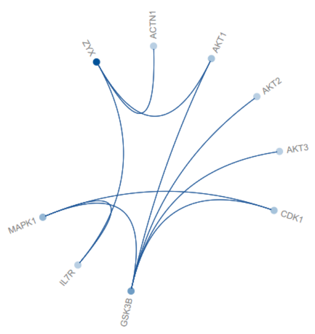

(B) STITCH Analysis

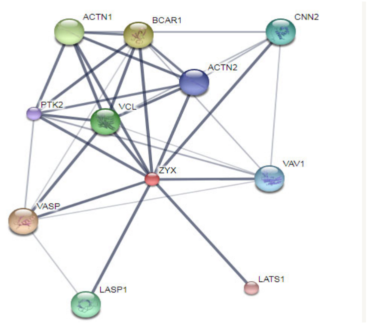

(C) STRING Analysis

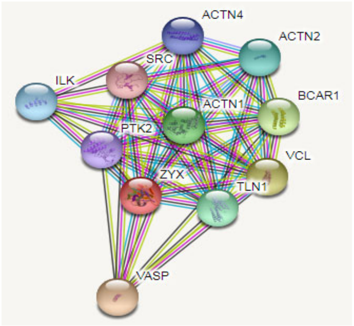

Supplementary Figure 15. Protein-protein interactions and pathway analysis, (A) Omnipath showing the zyxin involvement in Akt/IL7R pathway; (B) STITCH analysis; (C) STRING analysis.

Supplementary Table 1. Distribution of hydrophobic amino acids in each LIM domain of human Zyxin family proteins. The column for each LIM domain shows number of hydrophobic amino acids and the total hydrophobicity. These values were determined using Expasy ProtParam, the hydrophobicity of each domain varies from ~36 to 47 %. The number of hydrophobic amino acid varied in each domain but tryptophan was absent amongst all LIMs except LIM3 of TRIP6.

**Human Zyxin Family**	**Lim1**			**Lim2**			**Lim3**		
**Zyxin**	Ile=1	Phe=3	41.8%	Ile=2	Phe=2	36.6%	Ile=2	Phe=2	39.1%
	Val=1	Trp=0		Val=4	Trp=0		Val=5	Trp=0	
	Leu=6			Leu=2			Leu=4		
	Cys=7			Cys=6			Cys=8		
	Met=0			Met=1			Met=2		
	Ala=7			Ala=5			Ala=4		
**LimD1**	Ile=0	Phe=7	46.8%	Ile=4	Phe=3	47.6%	Ile=2	Phe=1	36.1%
	Val=4	Trp=0		Val=5	Trp=0		Val=4	Trp=0	
	Leu=3			Leu=6			Leu=7		
	Cys=7			Cys=7			Cys=8		
	Met=1			Met=2			Met=1		
	Ala=7			Ala=2			Ala=2		
**Ajuba**	Ile=2	Phe=4	40.3%	Ile=4	Phe=3	44.3%	Ile=3	Phe=2	43.3%
	Val=3	Trp=0		Val=5	Trp=0		Val=2	Trp=0	
	Leu=4			Leu=4			Leu=6		
	Cys=9			Cys=8			Cys=1		
	Met=0			Met=1			1		
	Ala=3			Ala=2			Met=3		
							Ala=3		
**LPP**	Ile=3	Phe=3	43.3%	Ile=5	Phe=4	44.3%	Ile=4	Phe=1	38.9%
	Val=5	Trp=0		Val=3	Trp=0		Val=5	Trp=0	
	Leu=2			Leu=3			Leu=5		
	Cys=8			Cys=6			Cys=8		
	Met=1			Met=2			Met=1		
	Ala=4			Ala=4			Ala=3		
**WTIP**	Ile=3	Phe=4	40.4%	Ile=3	Phe=3	46%	Ile=2	Phe=0	40.4%
	Val=2	Trp=0		Val=7	Trp=0		Val=3	Trp=0	
	Leu=4			Leu=4			Leu=8		
	Cys=7			Cys=7			Cys=9		
	Met=1			Met=2			Met=1		
	Ala=4			Ala=2			Ala=5		
**FBLIM**	Ile=1	Phe=3	37.1%	Ile=3	Phe=5	46.6%	Ile=5	Phe=3	40%
	Val=2	Trp=0		Val=4	Trp=0		Val=3	Trp=0	
	Leu=4			Leu=3			Leu=4		
	Cys=7			Cys=7			Cys=9		

	Met=1			Met=0			Met=1		
	Ala=5			Ala=5			Ala=3		
**TRIP6**	Ile=0	Phe=3	47.4%	Ile=5	Phe=4	45.3%	Ile=5	Phe=1	43.2%
	Val=9	Trp=0		Val=3	Trp=0		Val=3	Trp=1	
	Leu=2			Leu=3			Leu=6		
	Cys=7			Cys=6			Cys=9		
	Met=0			Met=1			Met=1		
	Ala=6,			Ala=5			Ala=4		

Supplementary Table 2. Ohnolog analyis of Zyxin family proteins.

Family Size	Ohnolog Family
3	AJUBA, LIMD1, WTIP
4	FBLIM1,LPP,TRIP6,ZYX

Supplementary Table 3. List of all online servers/databases used in the study.

Name of Softwares/Databases	Information
Uniprot	https://www.uniprot.org/
Muscle	https://www.ebi.ac.uk/Tools/msa/muscle/
ProtParam	https://web.expasy.org/protparam/
ProtScale	https://web.expasy.org/protscale/
NetNES	www.cbs.dtu.dk/services/NetNES/
NLStradamus	http://www.moseslab.csb.utoronto.ca/NLStradamus/
MultAlin	http://www.sacs.ucsf.edu/cgi-bin/multalin.py
PhosphoSitePlus v6.5.9.1	https://www.phosphosite.org/homeAction
Swiss model workspace	http://swissmodel.expasy.org/workspace/
Procheck analysis	https://servicesn.mbi.ucla.edu/PROCHECK/
Dali server	http://ekhidna2.biocenter.helsinki.fi/dali/
Bioplex explorer	https://bioplex.hms.harvard.edu/explorer/home
Pharos interface	https://pharos.nih.gov/
OmniPath	http://omnipathdb.org/
Signor 2.0	https://signor.uniroma2.it/
Genemania	https://genemania.org/
STRING analysis	https://string-db.org
STITCH analysis	http://stitch.embl.de/cgi/
Molecular INTeraction database (MINT)	https://mint.bio.uniroma2.it/
GENEVESTIGATOR	https://genevestigator.com/
iMODS server	http://imods.chaconlab.org/
WEBLOGO	https://weblogo.berkeley.edu/logo.cgi
OHNOLOGS v2	http://ohnologs.curie.fr
PhyML 3.0	http://www.atgc-montpellier.fr/phyml/
